# Supplementary figures and images for: Effects of polyploidization and their evolutionary implications are revealed by heritable polyploidy in the haplodiploid wasp Nasonia vitripennis
Source: PLoS One. 2023 Nov 2;18(11):e0288278. doi: 10.1371/journal.pone.0288278 (PMC10621845; doi:10.1371/journal.pone.0288278)

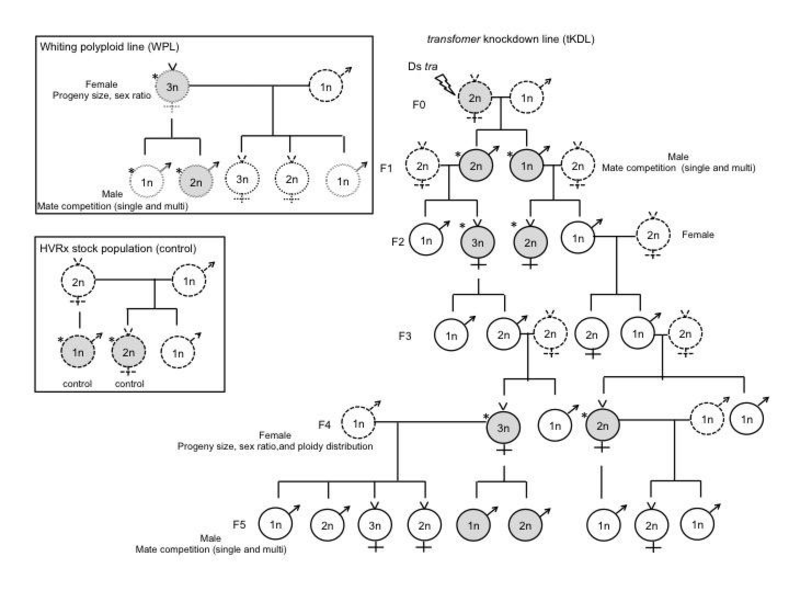

Supplement: S1 Fig — Horizontal lines indicate a cross and vertical lines indicate descent. Gray-filled symbols represent individuals that were used in assays. The untreated HVRx strain was used to generate control individuals for each generation and to continue breeding in the injected line. This background is represented with dashed lines. The transformer knockdown line (tKDL) was founded with F0 females injected with ds tra RNA. tKDL polyploids individuals from the F2 (female) and F3 (male) generations were not used for assays but were used to continue the line. The Whiting polyploid line (WPL) was used to produce inbred individuals of a long-established polyploid background to compare against outbred tKDL counterparts and is indicated by dotted lines. Individuals that were used for qPCR analysis of reference housekeeping genes Ak3 and ef1α are marked with an asterisk (*). (TIF) [file pone.0288278.s001.tif]

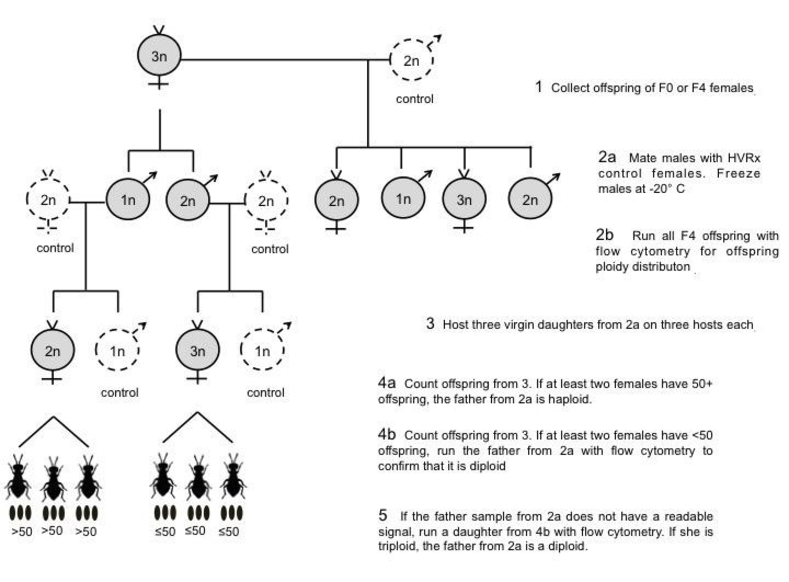

Supplement: S2 Fig — Horizontal lines indicate a cross and vertical lines indicate descent. Gray-filled symbols indicate individuals that were used in assays. The HVRx stock population background is denoted by dashed lines. The tKDL background is represented by solid lines. Ploidy was known a priori for WPL and control (HVRx) individuals. Typing for ploidy took place for tKDL haploid and diploid males as they have no distinguishing morphological markers. Ploidy typing of F1, F3, and F5 males used a two-step daughter-typing and flow cytometry approach. Ploidy could be inferred for F2 and F4 females through the ploidy of their corresponding fathers from the previous generation. The daughter-typing step is based on the lesser fecundity of triploid females. In a pilot study, control diploid HVRx females were capable of producing 60–90 offspring on three Calliphora sp. hosts. In contrast, triploid females of the WPL typically produce only four offspring (this paper). Hence, male ploidy can be partially determined from the fecundity of their daughters. For ploidy typing of the males, each male was mated to a virgin diploid HVRx female from the stock population. If the male was haploid, it produced diploid female offspring. If the male was diploid, it produced triploid female offspring. Three daughters of each male were hosted on three hosts each (to account for poor reproduction of random females), and the offspring allowed to develop under standard conditions. For each male, if at least two of the three daughters produced over 50 offspring each, they were scored as diploid females, and their father assigned corresponding haploid status. If all three daughters produced 0–50 offspring, this possibly reflected lower fecundity of triploid daughters. If the daughters of a male were scored as possible triploids, either the males themselves or one of their representative daughters were processed with flow cytometry. Flow cytometry samples were prepared by removing the head from the body, placing it [file pone.0288278.s002.tif]
