# Supplementary material for: Effects of polyploidization and their evolutionary implications are revealed by heritable polyploidy in the haplodiploid wasp Nasonia vitripennis
Source: PLoS One. 2023 Nov 2;18(11):e0288278. doi: 10.1371/journal.pone.0288278 (PMC10621845; doi:10.1371/journal.pone.0288278)
Supplement: S1 Table — (DOCX) [file pone.0288278.s003.docx]

**Table S1. Primers used for qPCR of genes *Ak3* and *ef1α*.**

| Gene | NCBI ref. seq | Forward primer | Reverse primer |
| --- | --- | --- | --- |
| *Ak3* | XM_016986045 | 5’-AATTCAATCG  GGTTCTGCTC-3’ | 5’-CAGCATCTCATC  TAACTTCTCTCTG-3’ |
| *ef1α* | XM_008209960 | 5’-CACTTGA TCT  ACAAATGCGGTG-3’ | 5’-CCTTCAGTTTGT  CCAAGACC-3’ |
